# Increase in anti-apoptotic molecules, nucleolin, and heat shock protein 70, against upregulated LRRK2 kinase activity

**DOI:** 10.1080/19768354.2018.1518262

**Published:** 2018-09-12

**Authors:** Jihoon Jang, Hakjin Oh, Daleum Nam, Wongi Seol, Mi Kyoung Seo, Sung Woo Park, Hyung Gun Kim, Hyemyung Seo, Ilhong Son, Dong Hwan Ho

**Affiliations:** a Department of Molecular and Life Sciences, Hanyang University, Ansan-si, Republic of Korea; b InAm Neuroscience Research Center, Sanbon Medical Center, College of Medicine, Wonkwang University, Gunpo-si, Republic of Korea; c Paik Institute for Clinical Research, Inje University College of Medicine, Busan, Republic of Korea; d Department of Health Science and Technology, Graduate School of Inje University, Busan, Republic of Korea; e Department of Pharmacology, College of Medicine, Dankook University, Cheonan-si, Republic of Korea; f Department of Neurology, Sanbon Medical Center, College of Medicine, Wonkwang University, Gunpo-si, Republic of Korea

**Keywords:** Parkinson’s disease rotenone leucine-rich repeat kinase 2 (LRRK2) nucleolin heat shock protein 70

## Abstract

Leucine-rich repeat kinase 2 (LRRK2) is involved in Parkinson’s disease (PD) pathology. A previous study showed that rotenone treatment induced apoptosis, mitochondrial damage, and nucleolar disruption via up-regulated LRRK2 kinase activity, and these effects were rescued by an LRRK2 kinase inhibitor. Heat-shock protein 70 (Hsp70) is an anti-oxidative stress chaperone, and overexpression of Hsp70 enhanced tolerance to rotenone. Nucleolin (NCL) is a component of the nucleolus; overexpression of NCL reduced cellular vulnerability to rotenone. Thus, we hypothesized that rotenone-induced LRRK2 activity would promote changes in neuronal Hsp70 and NCL expressions. Moreover, LRRK2 G2019S, the most prevalent LRRK2 pathogenic mutant with increased kinase activity, could induce changes in Hsp70 and NCL expression. Rotenone treatment of differentiated SH-SY5Y (dSY5Y) cells increased LRKK2 levels and kinase activity, including phospho-S935-LRRK2, phospho-S1292-LRRK2, and the phospho-moesin/moesin ratio, in a dose-dependent manner. Neuronal toxicity and the elevation of cleaved poly (ADP-ribose) polymerase, NCL, and Hsp70 were increased by rotenone. To validate the induction of NCL and Hsp70 expression in response to rotenone, cycloheximide (CHX), a protein synthesis blocker, was administered with rotenone. Post-rotenone increased NCL and Hsp70 expression was repressed by CHX; whereas, rotenone-induced kinase activity and apoptotic toxicity remained unchanged. Transient expression of G2019S in dSY5Y increased the NCL and Hsp70 levels, while administration of a kinase inhibitor diminished these changes. Similar results were observed in rat primary neurons after rotenone treatment or G2019S transfection. Brains from G2019S-transgenic mice also showed increased NCL and Hsp70 levels. Accordingly, LRRK2 kinase inhibition might prevent oxidative stress-mediated PD progression.

**Abbreviations:** 6-OHDA: 6-hydroxydopamine; CHX: cycloheximide; dSY5Y: differentiated SH-SY5Y; g2019S tg: g2019S transgenic mouse; GSK/A-KI: GSK2578215A kinase inhibitor; HSP70: heat shock protein 70; LDH: lactose dehydrogenase; LRRK2: leucine rich-repeat kinase 2; MPTP: 1-methyl-4-phenyl-1,2,3,6-tetrahydropyridine; myc-GS LRRK2: myc-tagged g2019S LRRK2; NCL: nucleolin; PARP: poly(ADP-ribose) polymerase; PD: Parkinson’s disease; PINK1: PTEN-induced putative kinase 1; pmoesin: phosphorylated moesin at t558; ROS: reactive oxygen species

## GRAPHICAL ABSTRACT

**Figure:**
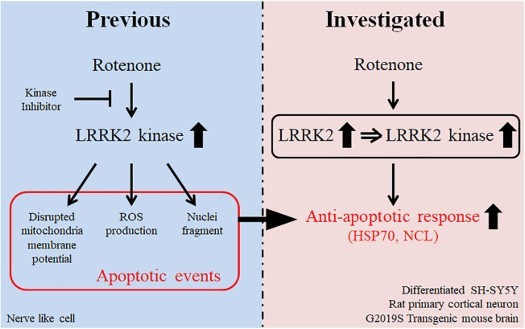

## Introduction

Parkinson’s disease (PD) is the second most common neurodegenerative disease. Loss of dopaminergic (DA) neurons in substantia nigra pars compacta is a pathological marker of PD. The degeneration of DA neurons is induced by several genetic factors such as mutations of α-synuclein, leucine rich-repeat kinase 2 (LRRK2), DJ-1, parkin, and PTEN-induced putative kinase 1 (PINK1), as well as chemical toxins including rotenone, 1-methyl-4-phenyl-1,2,3,6-tetrahydropyridine (MPTP), and 6-hydroxydopamine (6-OHDA). These PD-inducing factors are closely related with oxidative stress. DJ-1 is the molecular chaperone for reactive oxygen species (ROS) (Mitsumoto & Nakagawa 2001), and parkin and PINK1 are related to mitochondrial dynamics, which is sensitive to oxidative stress (Xiao et al. 2017). Accumulation of α-synuclein or generation of toxic α-synuclein oligomers has also been associated with producing ROS, ROS-mediated apoptosis, and defective mitochondria (Hsu et al. 2000; Tanaka et al. 2001). Finally, LRRK2 mutations, resulting in changes in kinase or GTPase activities, mediate oxidative stress and ROS-induced neuronal damage (Heo et al. 2010; Bahnassawy et al. 2013; Saez-Atienzar et al. 2014).

PD-linked chemical toxins affect mitochondrial respiration and generate ROS. In particular, rotenone inhibits respiratory mitochondria complex 1, thereby exacerbating ROS accumulation in cells (Li et al. 2003). Accelerated ROS could deteriorate protein homeostasis, lipids peroxidation, and DNA damage (Dias et al. 2013). These features overlapped with effects of genetic factors of PD on the degeneration of DA neurons. Nucleolar stresses have been reported as a PD-related cellular symptom (Parlato & Liss 2014), and ROS-mediated DNA damage is a culprit of nucleolar stress (Baltanás et al. 2011). A previous study revealed that nucleolin (NCL), a component of the nucleolus, is recruited to sites of DNA damage (Kobayashi et al. 2012; Goldstein et al. 2013). Another study demonstrated a reduction of NCL in the brains of PD patients. Interestingly, ectopic overexpression of NCL showed a neuroprotective role against rotenone treatment, but repression of NCL by siRNA aggravated rotenone-mediated neurotoxicity (Caudle et al. 2009). Heat shock protein 70 (HSP70), an anti-oxidative stress chaperone, is associated with neuronal protection against rotenone, MPTP, or toxic α-synuclein (Zhou et al. 2004; Dong et al. 2005). Silencing of HSP70 deteriorated 6-OHDA induced apoptosis in PC12 cells, a rat DA cell line (Alani et al. 2015).

H_2_O_2_ treatment to HEK293T cells expressing wild-type and G2019S LRRK2 induced LRRK2 kinase activity (Yang et al. 2012), and infusion of 6-OHDA in the mouse striatum induced LRRK2 mRNA and protein levels (Priya Nagappan 2015). Moreover, rotenone enhanced pS935 LRRK2, exacerbated mitochondria membrane potential, ROS production, and nuclei fragmentation in nerve-like cells, and these phenotypes were alleviated by PF-06447475, an LRRK2 kinase inhibitor (Mendivil-Perez et al. 2016). Thus, these results suggest that rotenone could increase LRRK2 levels or its kinase activity, thereby mediating anti-oxidative stress responses, such as the induction of NCL and HSP70, in DA neuron. To verify the role of LRRK2 kinase activity in rotenone-induced apoptosis, we performed a rotenone treatment or transfected G2019S LRRK2 with or without GSK2578215A, an LRRK2 kinase inhibitor, to differentiated SH-SY5Y (dSY5Y) neurons, an artificial human DA neuron cell line, and rat primary cortical neuron.

## Materials and methods

### Cell culture and treatment and lactate dehydrogenaseactivity (LDH) assay

For differentiation of human neuroblastoma SH-SY5Y cells (dSY5Y), cells were cultured in DMEM high glucose (Cellgro) with 10% FBS (Cellgro), 1% penicillin streptomycin (Gibco/BRL), and 10 μM all-trans retinoic acid for 7 days in a 5% CO_2_ incubator. At day 7, dSY5Y cells were treated with 0, 0.3, or 1 μM rotenone, or 1 μM rotenone with 25 μg/mL cycloheximide (CHX) for 24 h. Finally, cells were washed by ice-cold DPBS twice, and then whole cell lysates were obtained using 1X sample buffer. Cytotoxicity results of each chemical treatment or transfection were measured by LDH cytotoxicity detection kit (MK401, TAKARA) per the manufacturer’s instruction.

### Transfection and LRRK2 kinase inhibitor treatment

dSY5Y cells were transfected with 1.5 μg of LRRK2 G2019S mutant plasmid using Lipofectamine LTX reagent (Invitrogen) as per the manufacturer’s recommendation. Detailed information about LRRK G2019S mutant plasmid was previously provided (Shin et al. 2008). At 24 h post-transfection, we treated cells with 3 μM GSK2578215A (GSK/A-KI), an LRRK2 kinase inhibitor, for 24 h, and then whole cell lysates were obtained as previously described

### Western blot assay

For western blot assay, cell lysates were sonicated and boiled at 95°C for 5 min as previously described (Ho et al. 2015). All membranes were incubated with the following primary antibodies: anti-LRRK2 [N241A/34] (75-253, Neuromab), anti-LRRK2 pS935 (ab133450, Abcam), anti-LRRK2 pS1292 (ab230181, Abcam), anti-c-myc [9E10] (sc-40,Santa Cruz), anti-nucleolin (#14574, Cell Signaling Technology [CST]), anti-hsp70 (sc-66048, Santa Cruz), anti-moesin (sc-13122, Santa Cruz), anti-moesin pT558 (ab61109, Abcam), anti-PARP (9542S, CST), anti-α-tubulin (T9026, Sigma-Aldrich), and anti-β-actin (sc-47778, Santa Cruz). After primary antibody reaction, membranes were incubated in goat anti-rabbit or anti-mouse IgG with horseradish peroxidase (Jackson Immunoresearch). Intensities of the bands were detected by the Multi-Gauge V 3.0 program (Fuji photo Film).

### Rat primary cortical neuron isolation and culture

Isolation of rat primary neurons was performed as previously described (Seo et al. 2016), but cortical neurons instead of hippocampal neurons were collected. Culture conditions and media for cortical neurons are identical with that for hippocampal neurons. Animal experiments proceeded with the approval from the Committee for Animal Experimentation and the Institutional Animal Laboratory Review Board of Inje Medical College (approval No. 2016-044).

### Transgenic mouse brain preparation

G2019S TG mice were purchased from The Jackson Laboratory (strain B6; C3-Tg [PDGFB-LRRK2*G2019S] 340D jmo/J, stock number 016575) (Ramonet et al. 2011) and were housed in a specific pathogen-free facility at the Dankook University Animal Facility with the approval of the Dankook University Institutional Animal Care and Use Committee (DKU-16-035). G2019S mice and littermates were sacrificed by cervical dislocation. Brains were removed from the skull and lysed by ice-cold PBS with 1% Triton X-100 and 1Xprotease inhibitor cocktail (Calbiochem). Brain lysates were homogenized using a 17-gauge needle, incubated for 30 min at 4°C, and centrifuged at 4,000×*g* for 10 min at 4°C. Each supernatant was transferred to new tube and analyzed by western blotting.

### Statistical analysis

The graphs and statistical analysis were performed using Prism6 (GraphPad software). Data is presented as the mean ± SEM. Each statistical analysis is described in detail in the figure legends. Significances are presented as following: *p < .05; **p < 0.01; ***p < .001; and ****p < .0001.

## Results

### Rotenone increases LRRK2 levels and its kinase activity

**Figure 1.**
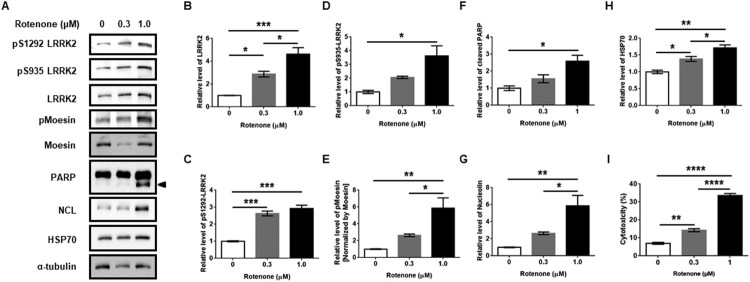
Rotenone treatment increased levels of LRRK2 protein and phosphorylated LRRK2. (A) Western blot analysis for dose-dependent treatment of rotenone in dSY5Y cells for 24 h. Arrowhead indicates cleaved PARP protein. (B–H) The graphs present relative densitometric levels of the proteins of interest, which was normalized to α-tubulin, except for pMoesin, which was normalized to total moesin. (I) Rotenone-induced dose-dependent cytotoxicity. One-way ANOVA with Tukey’s multiple comparison test.

To validate effects of rotenone on LRRK2 kinase activity, we treated differentiated human neuroblastoma SH-SY5Y (dSY5Y) cells, which were differentiated for 7 days with all-trans retinoic acid to exhibit DA neuronal-like properties, with rotenone in a dose-dependent manner. We observed that rotenone clearly increased LRRK2 protein levels, as well as pS935 and pS1292 LRRK2 signal in a dose-dependent manner. (Figure 1(A–D)). Moesin is an LRRK2 substrate, and T558 residue has been demonstrated to be the phosphorylation site of LRRK2 (Jaleel et al. 2007). Phosphorylated moesin at T558 (pMoesin) normalized by total moesin also showed a gradual increase in a dose-dependent manner due to rotenone treatment (Figure 1(A,E)). These results suggested that total LRRK2 kinase activity in dSY5Y was correlated with rotenone-derived cellular stress. In addition, apoptosis by rotenone treatment, represented by cleaved poly(ADP-ribose) polymerase (PARP) levels and lactose dehydrogenase (LDH) activities of culture media, were elevated with increasing LRRK2 kinase activity (Figure 1(A,F,I)). However, HSP70 and NCL, which are anti-apoptotic molecules, were activated against apoptotic damage of dSY5Y (Figure 1(G,H)). We hypothesized that a neuronal survival mechanism, such as the induction of anti-apoptotic factors, against rotenone, would be initiated by increased LRRK2 kinase activity.

### NCL and HSP70 are critical for the neuroprotection against rotenone and LRRK2 kinase activity

**Figure 2.**
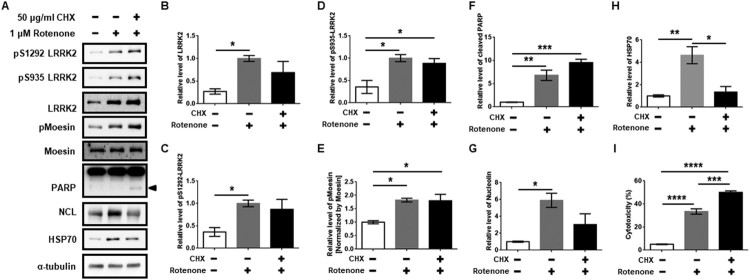
Inhibition of protein synthesis repressed induction of Nucleolin and HSP70 against rotenone. (A) Western blot analysis for co-treatment of CHX and rotenone in dSY5Y for 24 h. Arrowhead indicates cleaved PARP protein. (B–H) The graphs present relative densitometric levels of the proteins of interest. (I) Cytotoxicity of rotenone versus cycloheximide co-treatment with rotenone. The normalization and static analysis are the same as Figure 1.

We speculated that the blockage of NCL and HSP70 induction using cycloheximide (CHX), a protein synthesis inhibitor, could promote severe neuronal toxicity via repression of NCL and HSP70 proteins upon increased total LRRK2 kinase activity. As expected, we confirmed that CHX co-treatment significantly increased cytotoxicity as well as cleaved PARP (Figure 2(A,F,I)). Surprisingly, LRRK2 protein levels and its kinase activity after a co-treatment with CHX and rotenone were not reduced and still retained high levels compared to those of a rotenone only treatment (Figure 2(A–E)). However, increased NCL and HSP70 due to rotenone treatment were significantly reduced by co-treatment with CHX (Figure 2(A,G,H)). These results demonstrated that induction of NCL and HSP70 are critical for neuronal survival against rotenone-mediated neurotoxicity accompanied by increased LRRK2 kinase activity.

### Induced NCL and HSP70 levels by g2019S LRRK2 are repressed by the kinase inhibitor

**Figure 3.**
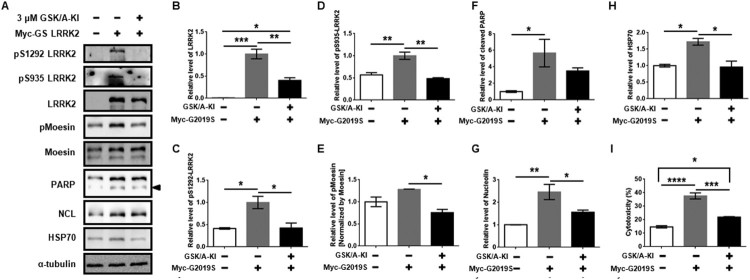
Changes in Nucleolin and HSP70 by ectopic myc-GS LRRK2 expression with or without LRRK2 kinase inhibitor. (A) Western blot analysis for transient expression of myc-tagged G2019S LRRK2 (myc-GS LRRK2) to dSY5Y for 48 h with or without the 24-h post-treatment of GSK2578215A (GSK/A-KI), an LRRK2 kinase inhibitor. Arrowhead indicates cleaved PARP protein. (B–H) All protein bands, except pMoesin, were normalized to α-tubulin, and estimated relative levels of the proteins of interest are presented. (I) Cytotoxicity of myc-tagged G2019S transfection with or without GSK2578215A. One-way ANOVA with Tukey’s multiple comparison tests.

In a previous study, the administration of LRRK2 kinase inhibitor rescued rotenone-mediated mitochondrial dysfunction, the increase in ROS levels and a fragment of the nucleus (Mendivil-Perez et al. 2016). Furthermore, we expected that an increase in NCL and HSP70 by ectopic G2019S LRRK2 expression would be decreased by an LRRK2 kinase inhibitor. G2019S LRRK2 is a well-known pathological mutation that harbors enhanced kinase activity. Therefore, we transfected myc-tagged G2019S LRRK2 (myc-GS LRRK2) to dSY5Y cells for 48 h, with a post-treatment of DMSO or GSK/A-KI for 24 h. We found that myc-GS LRRK2-derived induction of NCL and HSP70 were repressed by GSK/A-KI (Figure 3(A,G,H)), and cells also exhibited decreased cytotoxicity (Figure 3(I)). However, there was a slight decrease in cleaved PARP by GSK/A-KI compared to that of myc-GS LRRK2 (Figure 3(F)). Kinase activity of myc-GS LRRK2 and activity of GSK/A-KI were identified by pS935, pS1292, and pMoesin (Figure 3(A–E)). These results revealed that rotenone treatment enhanced total LRRK2 kinase activity as well as LRRK2 G2019S kinase activity, which would be able to activate NCL and HSP70 induction, therefore suggesting that the regulation of LRRK2 kinase activity was a key step for modulating apoptosis in dSY5Y cells.

### Induced NCL and HSP70 levels by rotenone-mediated LRRK2 activity or g2019S kinase activity in rat primary cortical neuron and transgenic mouse brain

**Figure 4.**
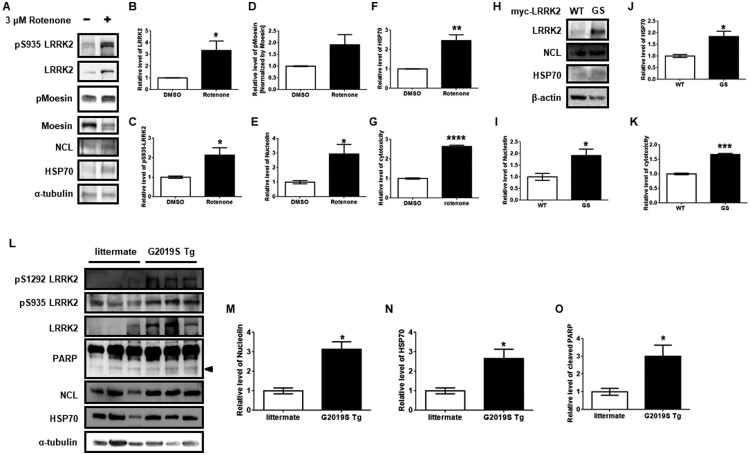
Increased Nucleolin and HSP70 in rat primary cortical neuron with rotenone treatment or transient expression of myc-GS LRRK2 and G2019S Tg mouse brain. (A) Western blot analysis for 3 μM rotenone treatment in rat primary cortical neuron for 24 h. (B–F) Each protein was normalized to α-tubulin, except for pMoesin, which was normalized to total moesin. (H) Two micrograms of myc-tagged WT LRRK2 (WT) or G2019S LRRK2 (GS) were transfected into rat primary cortical neuron for 24 h. (I and J) Densitometric analysis of each protein was normalized by β-actin. The cytotoxicity of rotenone treatment (G) and transiently expressing myc-tagged wild-type LRRK2 or LRRK2 G2019S (K) in rat primary cortical neurons. (L) Western blot analysis of brain lysates from G2019S LRRK2 transgenic mice (G2019S Tg) and littermates. (M–O) Levels of NCL, HSP70 and cleaved PARP (arrow head) were normalized by α-tubulin. Every statistical analysis was performed with Student’s *t*-test.

To verify similar phenotypes *ex vivo*, isolated rat primary cortical neurons were treated with rotenone for 24 h. Again, rotenone significantly increased NCL, HSP70, LRRK2, and pS935 levels, but increases in pMoesin were not significant (Figure 4(A–F)). Moreover, transient expression of myc-GS LRRK2 enhanced NCL and HSP70 protein compared to myc-WT LRRK2 (Figure 4(H–J)). Relative levels of cytotoxicity via an LDH activity assay revealed the elevation of neuronal toxicity due to rotenone treatment or transfection with myc-GS compared to myc-WT in rat primary cortical neuron (Figure 4(G,K)), supporting the hypothesis that induction of NCL and HSP70 confers protection against apoptosis. Surprisingly, the brain of G2019S LRRK2 transgenic mice (G2019S Tg) exhibited significantly higher levels of NCL and HSP70 than those of the non-Tg littermate (Figure 4(L–N)). Cleaved PARP was significantly increased in G2019S Tg mice compared to their littermates (Figure 4(O)). LRRK2, pS935, and pS1292 levels were higher in G2019S Tg mice compared to those of the non-Tg littermates (Figure 4(L)). Taken together, increased anti-apoptosis responses in G2019S Tg mice were due to upregulated LRRK2 kinase activity.

## Discussion

Oxidative stress-derived apoptosis has been hypothesized to contribute to PD progression (Puspita et al. 2017). Several studies of LRRK2 revealed that its kinase or GTPase activity contributes to oxidative stress and cytotoxicity in neurons (Heo et al. 2010; Bahnassawy et al. 2013; Saez-Atienzar et al. 2014). These phenotypes were associated with chemical toxins linked to PD, and mitochondrial damage has been reported not only in the G2019S LRRK2 model but also the rotenone-induced PD model (Li et al. 2003; Sanders et al. 2014). A previous study demonstrated that mitochondrial damage, generation of ROS, and DNA damage by rotenone could be mitigated by inhibition of LRRK2 kinase activity in nerve-like cells (Mendivil-Perez et al. 2016). A previous study reported that LRRK2 interacted with carboxyl terminus of the Hsc70-interacting protein (CHIP)/HSP70 complex, and CHIP attenuated LRRK2-mediated cytotoxicity via degradation of LRRK2 (Ko et al. 2009). Previous studies have also shown that enhancement of HSP70 by prostaglandin A1 and geldanamycin rescued rotenone- or MPTP-mediated cytotoxicity, respectively (Wang X et al. 2002; Shen et al. 2005). Gene transfer of HSP70 using viral vectors also reduced MPTP-derived DA neuronal loss (Dong et al. 2005). Our results demonstrated that rotenone-induced LRRK2 kinase activity was dependent on increased LRRK2 protein levels, and upregulation of total LRRK2 kinase activity mediated HSP70 induction. These results suggest that induced HSP70 against LRRK2 kinase activity might be one of the protective feedback strategies of cells to attenuate total LRRK2 kinase activity via degradation of LRRK2 protein by the CHIP/HSP70 complex. Therefore, overexpression of HSP70 might effectively attenuate LRRK2 kinase activity, which would then confer protection against cytotoxicity due to rotenone, thereby reducing DA neuronal damage.

Oxidative stress is also involved in DNA damage, and DNA damage has been reported as a marker of PD pathology (Alam et al. 1997). NCL has been demonstrated to be important for DNA repair and maintaining nucleolar structure (Kobayashi et al. 2012; Goldstein et al. 2013). Nucleolar fragmentation has also been reported to be an oxidative sensor in *S. cerevisiae* (Lewinska et al. 2010). Repression of NCL has been reported to be responsible for the disruption of the nucleolar structure and vulnerability to irradiation-mediated cell death (Ugrinova et al. 2007). In a previous study, brains of PD patients showed decreased NCL, and overexpression of NCL increased resistance against rotenone. However, repression of NCL resulted in greater vulnerability to rotenone (Caudle et al. 2009). Our data showed that treatment with rotenone alone increased NCL whereas co-treatment of rotenone with CHX resulted in reduced NCL and higher cytotoxicity. Taken together, an increase of NCL against rotenone may be an endogenous protective mechanism by inducing DNA repair and sustaining nucleolar structure against oxidative stress. In contrast to brains from PD patients, the G2019S Tg brain showed elevated NCL levels, but this may have been due to age of G2019S Tg mice. We used 18- to 26-week-old mice for this study (Figure 4(L)), but NCL levels were indistinguishable in the brains of mice older than 52 weeks (data not shown). These phenotypes might be due to the accumulation of ROS by aging in non-Tg littermate since oxidative stress is involved in aging. We speculated that dysregulated protein quality control in aging PD patients would affect degradation of NCL compared to our G2019S Tg mouse model.

Induction of HSP70 could also be involved in an NCL-mediated cellular protective mechanism, because induction of HSP70 and NCL are co-regulated during early stages of liver regeneration (Konishi et al. 1995). A previous study reported that H_2_O_2_-induced nucleolar fragmentation and NCL degradation in mouse embryonic fibroblasts were rescued by increasing HSP70 (Wang K et al. 2012). Another study demonstrated that transfected HSP70 reduced H_2_O_2_-induced apoptosis via stabilization of NCL, and NCL knockdown did not rescue H_2_O_2_-induced apoptosis even in HSP70 overexpressing cells (Jiang et al. 2010). These results would support the protective role of NCL and HSP70 against neuronal toxicity by LRRK2 kinase activity.

The role of G2019S in PD pathogenesis in generating cellular or clinical symptoms is still unclear. However, previous studies have revealed that G2019S LRRK2 alters various cellular homeostasis mechanisms, such as autophagy-lysosome pathway, vesicle trafficking, mitochondria dysfunction, and accumulation of ROS in immortalized cells, primary cells, or animal models (Shin et al. 2008; Heo et al. 2010; Ramonet et al. 2011; Saez-Atienzar et al. 2014; Mendivil-Perez et al. 2016). Strangely, LRRK2 G2019S mutation is known to contribute to late-onset PD despite increasing neuronal vulnerability by itself (Healy et al. 2008). Our results demonstrated that G2019S-mediated increase in HSP70 or NCL levels could result in resistance against apoptotic cell death, which might be derived by altered cellular homeostasis. These evidences suggest that induction of defensive machinery against altered cellular homeostasis in LRRK2 G2019S mutants may contribute to the late onset of PD. In previous studies, LRRK2 G2019S was found in sporadic PD patients (1–2%), and the penetration of G2019S was gradually increased from 28% in 59-year-old patients, to 74% in 79-year-old patients (Healy et al. 2008). Increases in LRRK2 kinase activity via accumulation of aging-mediated oxidative stress along with the LRRK2 G2019S mutant protein could result in neuronal death or degeneration because of harsher environmental conditions. Altogether, regulation of LRRK2 kinase against oxidative stress throughout life may be an effective approach for a preventive therapy against PD progression.

Herein, we confirmed that rotenone-mediated cytotoxicity was accompanied by LRRK2 kinase activity. Moreover, neuronal survival against oxidative stress derived by increased LRRK2 kinase activity could be facilitated via induction of HSP70 and NCL. Overall, a LRRK2 kinase inhibitor may be a better therapeutic option in preventing oxidative stress-induced apoptosis of DA neurons rather than simply rescuing damaged DA neurons via excessive expression of HSP70 or NCL.
